# Clinicopathological significance of stromal variables: angiogenesis, lymphangiogenesis, inflammatory infiltration, MMP and PINCH in colorectal carcinomas

**DOI:** 10.1186/1476-4598-5-43

**Published:** 2006-10-06

**Authors:** Xiao-Feng Sun, Hong Zhang

**Affiliations:** 1 Department of Oncology, Institute of Biomedicine and Surgery, University of Linköping, SE-581 85 Linköping, Sweden; 2 Department of Dermatology, Institute of Biomedicine and Surgery, University of Linköping, SE-581 85 Linköping, Sweden

## Abstract

Cancer research has mainly focused on alterations of genes and proteins in cancer cells themselves that result in either gain-of-function in oncogenes or loss-of-function in tumour-suppressor genes. However, stromal variables within or around tumours, including blood and lymph vessels, stromal cells and various proteins, have also important impacts on tumour development and progression. It has been shown that disruption of stromal-epithelial interactions influences cellular proliferation, differentiation, death, motility, genomic integrity, angiogenesis, and other phenotypes in various tissues. Moreover, stromal variables are also critical to therapy in cancer patients. In this review, we mainly focus on the clinicopathological significance of stromal variables including angiogenesis, lymphangiogenesis, inflammatory infiltration, matrix metalloproteinase (MMP), and the particularly interesting new cysteine-histidine rich protein (PINCH) in colorectal cancer (CRC).

## Background

The majority of cancer researchers have been focusing on tumour cells themselves and investigating their alterations in morphology, biology and function in tumour processes. However, stromal variables within or around tumours, including blood and lymph vessels, stromal cells, and various types of proteins, have not drawn enough attention even though they have important impacts on tumour development and progression. Tumour angiogenesis and lymphangiogenesis are the processes of creating new blood vessels or lymph vessels within and surrounding tumours. Stromal cells consist of various cell types such as infiltrating immune cells, fibroblasts, and endothelial cells. The extracellular matrix (ECM) is a complex structural entity surrounding tumour cells, and is often referred to as the connective tissue or ground substance. The ECM is composed of three major classes of biomolecules; structural proteins (collagen and elastin), specialized proteins (fibrillin, fibronectin, and laminin), and proteoglycans [1,2].

A better understanding of the role of stromal variables in tumour development is required for designing appropriate therapeutic strategies against angiogenesis and stromal proteinases. A number of anti-angiogenesis elements and matrix metalloproteinase (MMP) inhibitors (MMPI) have recently been developed, and some have reached clinical trials [3-7]. Compared to tumour cells, stromal variables are more attractive therapeutic targets, due to lower drug resistance and few side effects [8].

In this article, we mainly review the clinicopathological significance of stromal variables, including angiogenesis, lymphangiogenesis, inflammatory infiltration, MMPs and the particularly interesting new cysteine-histidine rich protein (PINCH) in colorectal cancer (CRC).

### Angiogenesis and lymphangiogenesis in CRCs

In general, normal tissues have a barrier, preventing either endothelial cell migration or tumour cell invasion. The effect of this barrier can be interrupted by newly-formed stroma, namely stromatogenesis, during the process of tumour development [9]. Stromatogenesis is probably a response to messages delivered by tumour cells. The newly-formed stroma is usually loose and oedematous and therefore allows endothelial and tumour cells to easily penetrate it [10].

When a tumour grows larger than 1–2 mm^3^, it must stimulate the host to creat its own vasculature to be able to continue growing. To accomplish this process, tumour cells induce adjacent blood vessels to sprout new vessels toward the tumour in a process called tumour angiogenesis [11]. Since immature microvessels are not covered by pericyte, and they are irregular and leaky tumour cells can more easily penetrate immature microvessels than mature microvessels [12]. Lymphangiogenesis is the process of creating new lymph vessels within or surrounding a tumour. As compared with blood capillaries, lymphatic endothelial cells have even poorly developed junctions with frequently large inter-endothelial gaps. In addition, lymphatic vessels have discontinuous or completely absent basement membranes [13-15]. Lymphangiogenesis is a relatively new area of basic and clinical investigation, and has not been well studied due to a lack of specific lymphatic vessel markers. The recent discovery of specific lymphatic vessel markers, and their corresponding antibodies have aided in the identification of lymphatic vessels. Importantly, increased interest in this field has been generated by the discovery of new vascular endothelial growth factor (VEGF) family members which play a critical role in lymphangiogenesis [14].

#### Growth factors in angiogenesis and lymphangiogenesis

Both physiological and pathological stimuli such as hypoxia induce tumour cells, together with leukocytes, macrophages, mast cells and platelets, to secrete VEGF and other growth-related factors such as platelet-derived growth factor (PDGF) family proteins and their receptors (PDGFR), insulin-like growth factor (IGF) proteins and their receptors (IGFR), circulating endothelial precursor cell (CEPC), fibroblast growth factors (FGF)-2, angiopoietins, EphrinB2 and EphB4 [13]. These factors are then directly or indirectly involved in angiogenesis and lymphangiogenesis, causing endothelial cells of nearby blood and lymph vessels to divide, migrate and form new vessels growing toward the tumour. Among these factors, the VEGF family is the best characterised.

The ligands of the VEGF family include VEGF-A, VEGF-B, VEGF-C, VEGF-D and VEGF-E. All five ligands have different roles in the process of angiogenesis and lymphangiogenesis. Each of the VEGF-family ligands binds to one or more of three known VEGF receptors (VEGFR): VEGFR-1 (also known as flt-1), VEGFR-2 (Flk-1 or KDR) and VEGFR-3 (Flt-4). VEGFR-1 organises blood vessels, and has a high affinity for VEGF-A and VEGF-B. VEGFR-2 activates blood vessel proliferation by binding to VEGF-A, VEGF-C, VEGF-D, and VEGF-E. VEGFR-2 is expressed in lymphatic endothelial cells [13,16-18]. VEGFR-3 is expressed on the vascular endothelium, but is mainly restricted to the lymphatic endothelium. VEGFR-3 binds to VEGF-C and VEGF-D, and is critical to the growth migration and survival of lymphatic endothelial cells, resulting in lymphangiogenesis [19-24].

#### Angiogenesis in relation to clinicopathological variables

It has been found that high microvessel density (MVD) is associated with VEGF [25-27] and VEGF-C expression at the deepest invasive tumour site [24,28]. However, high MVD is not associated with VEGF-D expression [24]. Several studies have demonstrated that MVD gradually increases from normal mucosa to adenoma and finally to carcinoma in the colorectum [29-33]. Increased MVD is detected at the early stages of focal dysplasia, and then increases gradually from low to high grades of dysplasia [33]. The transitional mucosa adjacent to the carcinoma displays intermediate levels of MVD between normal mucosa and the carcinoma [29]. In the carcinoma, MVD increases as the tumour invades from the mucosa to the muscularis propria [32]. The highest level of MVD is found at the invasive margin of carcinomas [34], a site of active tumour invasion. These findings indicate that MVD is an early and critical step in colorectal tumourigenesis and tumour development.

Many research groups have studied CRC and shown that a high grade of MVD is related to a larger tumour size [12,35,36], non-mucinous carcinoma [27], poorer differentiation [12,34], deeper invasion of tumours [12], advanced Dukes' stage [12,34], lymphatic vessel invasion [34-36], lymph node metastasis [31,34,36], venous vessel invasion [34-37], liver metastasis [34], and a higher rate of recurrence [36,38-40]. Tumours with high levels of MVD have been connected to poor survival in patients with earlier or advanced colon/rectal cancers [12,27,40-42]. Even in multivariate analyses, MVD is related to survival in the whole group of patients with CRC [34,36,38,43] or subgroups of patients with stage II-III [44] or stage A-C tumours [37]. Recently, Yonenaga *et al*. have analysed a microvessel pericyte coverage index (an index of microvessel maturation) in relation to clinicopathological significance in CRC [12]. The results indicate that immature neovascularization is observed in poorly differentiated tumours and further correlated with metastasis, resulting in a poorer prognosis. Thus, not only microvessel density but also vessel maturation are crucial factors for the tumour development and aggressiveness of CRC. Notably, Yonenaga *et al*. have applied the anti-α-smooth muscle actin (SMA) marker to determine the microvessel pericyte coverage index [12]. There are several other common markers used for pericytes including desmin, PDGFR-β, VEGFR-1, and neuron-glial antigen 2. It seems that α-SMA and desmin expression are esentially identical in pericytes [12,45]. While PDGFR-β, VEGFR-1, and neuron-glial antigen 2 can be positively expressed in pericytes that are negative for α-SMA, indicating that a lack of α-SMA expression does not necessarily mean an absence of pericytes. In other words, PDGFR-β, VEGFR-1, and neuron-glial antigen 2 may be more reliable markers for determining the presence of pericytes, while α-SMA is probably only expressed in more stable and mature pericytes [46].

There are a few conflicting reports in CRC regarding the clinicopathological significance of MVD, in which MVD is not associated with tumour stage [29,43,47], vascular and neural invasion [47], metastasis [29], or survival in the whole group of patients [4,29,48,49], subgroups of patients in stage A-C [47], or patients with stage I and II rectal cancer [50]. In addition, there were four studies which are performed on a small number of colon and/or rectal cancers (from 22 to 48 cases) that also show a non-association of MVD with the clinicopathological variables including tumour size, location, grade of differentiation, the presence of a mucinous component, stage, vascular or lymphovascular or neural invasion, or patient survival [30,51-53].

There are even opposite results from two studies in which higher values of MVD appear to be in the early stages of CRC [54], and correlate with longer disease-free survival and overall survival in patients with node-negative CRC [55]. Recently, Peeters *et al*. observed an increased vascularization of metastases in the liver after resection of the primary CRC [56]. This result suggests that the primary tumour may produce certain circulating inhibitors of angiogenesis that suppress the angiogenesis of metastases. Therefore, after resection of the primary tumour, the circulating levels of this inhibitor decrease, resulting in increased angiogenesis and, as a consequence, growth of the metastases [57].

Hypercoagulation in cancer patients is another factor for tumour progression. Substantial evidence from preclinical experiments and clinical practice has supported the association between activation of blood coagulation and progression of the cancer. Cancer patients display a wide range of coagulation disorders from asymptomatic laboratory changes to massive thromboembolism and disseminated intravascular coagulation. About 50% of all cancer patients and 90% of patients with metastasis have abnormalities in coagulation tests. CRC is the second most common cancer diagnosed in patients with thromboembolic events. Blood vessel thrombosis leads to impairment of blood flow, ischemia, and organ damage. The hemostatic complications are the second most common cause of death in cancer patients [58].

Studies have shown that plasma D-dimer levels, representing activation of coagulation and fibrinolysis, are increased in most patients with CRC compared to patients with benign colorectal disease. Furthermore, the D-dimer level is positively related to tumour size, wall penetration, lymph node invasion, and hepatic metastasis [59]. In a multivariate analysis, the D-dimer level in preoperative plasma in CRCs is the third strongest prognostic factor, after lymph node status and preoperative carcinoembryonic antigen level [60]. Fibrinolytic capacity was much higher in advanced CRCs, indicating a progression to overt disseminated intravascular coagulation [58].

Although the mechanism behind hypercoagulation in cancer patients is unclear, the main factor responsible for hypercoagulation has been considered to be cancer itself. It has been shown that tumour cells activate the coagulation system by producing and secreting procoagulant/fibrinolytic substances and inflammatory cytokines, as well as physically interacting with blood (monocytes, platelets, neutrophils) or vascular cells [61]. This activation is accompanied by the consumption and decline of coagulation inhibitors. Other mechanisms for hypercoagulation in cancer patients include non-specific factors such as the generation of acute phase reactants, necrosis, abnormal protein metabolism and hemodynamic compromise. In addition, anticancer therapy may also increase the risk of blood coagulation by similar mechanisms, e.g., release of procoagulant/fibrinolytic substances and inflammatory cytokines, damage of endothelial cells, and stimulation of tissue factor production by host cells [61].

A recent study shows that VEGF is highly expressed in primary CRC compared to the corresponding adjacent normal mucosa [62]. VEGF expression appears to be absent in mild to moderate dysplasia adenomas of the colorectum, and is present in the majority of carcinomas-in-situ and in all carcinomas invading the submucosa [32]. VEGF-D is more highly expressed in carcinoma than in the adjacent normal mucosa [22,24] and adenoma [24], while VEGF-C expression in normal mucosa does not differ from that in CRC [22]. Notably, one study shows that VEGF-D expression is significantly lower in both polyp and carcinoma compared to normal mucosa while VEGF-A and VEGF-C are significantly raised in carcinoma compared to normal mucosa and polyp. One explanation for this is that decreased VEGF-D may allow for higher levels of VEGF-A and VEGF-C to bind more readily to the VEGF receptors, producing the angiogenic switch required for tumour growth [19].

Increased expression of VEGF-A in CRC is associated with lymphatic metastases [19]. Increased VEGF-C expression correlates significantly with poorer differentiation [28], deeper invasion of tumours [28,63], advanced Duke's stage [28], lymphatic invasion, lymph node metastasis [28,63], venous invasion [28], and liver metastasis [28,64]. VEGF-D is associated with lymphatic involvement [24]. Overall, high VEGF expression is related to larger tumour size [65,66], non-mucinous carcinoma [27], advanced stage [65-67], blood vessel invasion, liver metastasis [67], multiple numbers of metastases [55], and recurrence [68].

There are few studies of VEGFR expression in CRC. Some studies show that either VEGFR-2 or -3 expression on CRC does not differ from that in the normal mucous of the colorectum [19,22], while others show that VEGFR-3-positive vessel densities increase progressively from normal mucosa to adenoma and to carcinoma [22,24,69]. Furthermore, VEGFR-3 is associated with lymph node metastasis [69].

There are controversial results regarding the role of VEGF and VEGFR in CRC. For example, levels of VEGF expression in primary CRC and liver metastases do not significantly differ [55]. VEGF-A has no impact on patient survival [4]. VEGF-C is not related to gender, histological type, venous involvement [63], lymph node invasion [19], liver metastasis or survival [63]. VEGF-D and VEGFR-3 expression do not correlate with grade of differentiation, Dukes' stage (A to C) or survival [24].

Even splicing variants in certain members of the VEGF family play different roles in tumour development. For example, the VEGF-A gene, located on chromosome 6p21.3 with eight exons, gives rise to several distinct isoforms of VEGF-A through alternative mRNA splicing. The more common isoforms of human VEGF-A consist of VEGF121, VEGF145, VEGF165, VEGF165b, VEGF189, and VEGF206, and other isoforms such as VEGF148, VEGF162 and VEGF183 have also been reported. VEGF-B also has different isoforms such as VEGF167 and VEGF186 [70,71]. These isoforms differ in their expression patterns as well as their biochemical and biological properties. In normal colonic tissue, VEGF121 and VEGF165 are mainly expressed, whereas VEGF189 is expressed rarely and weakly. VEGF121 and VEGF165 are diffusible secreted proteins with low affinity to heparin, whereas VEGF189 and VEGF206 have a high affinity to heparin-like molecules such as heparansulfate [72]. Okamoto *et al*. examined the expression patterns of several VEGF-A isoforms in 228 established xenografts originating from various human solid tumours including colon cancer. VEGF121/VEGF165 were seen in 27 xenografts and VEGF121/VEGF165/VEGF189 in 201 xenografts. VEGF189 is more frequently expressed in all tumour xenografts than in primary tumours, indicating that VEGF189 contributes to the successful xenotransplantability of various solid tumours through the induction of stromal vascularization [73]. Although the rate of tumour growth depends on the level of VEGF expression, certain isoforms play a greater role in angiogenesis than others. VEGF165b inhibits VEGF165-mediated proliferation, migration of endothelial cells, and vasodilatation of mesenteric arteries. VEGF165b-expressing tumours grow significantly more slowly than VEGF165-expressing tumours. Thus, VEGF165b is an effector of anti-angiogenesis and is downregulated in certain tumours. These results suggest that regulation of VEGF splicing is a critical switch from an antiangiogenic to proangiogenic phenotype [74,75].

#### Lymphangiogenesis in relation to clinicopathological variables

There are only a few studies of lymphatic density in CRC. Parr and Jiang examined lymph vessel status by using several lymphangiogenic markers (LYVE-1, Prox-1, podoplanin and 5'-nucleotidase), and found that their expression was higher in CRC compared to normal mucosa [22]. In adenoma, lymphatic vessels in stalk stroma were closely associated with early invasive epithelial nests [76]. Recently, Kuroyama *et al*. observed that intratumoural lymphatic vessels were present in the majority of colon carcinomas (91%), and had a significantly higher density in the submucosa near the tumour [77]. Furthermore, intratumoural lymphatic density is positively related to lymph node metastasis and arteriolar density, but not to tumour size, depth of tumour invasion, distant metastasis or TNM stage [77].

Like new blood vessels, lymphatics at the centre of tumours do not function as well in spreading tumour cells as they do at the invasive margin of tumour. The intratumoural lymph vessels are often compressed and smaller, while the lymph vessels around tumours are often enlarged and hyperplastic. These enlarged vessels may collect tumour cells from the tumour and possibly contribute to lymphatic metastasis. VEGF-C and VEGF-D induce not only the density but also the enlargement of lymphatic vessels, which leads to metastases to the regional lymph nodes [78-80].

Anginogenesis, lymphangiogenesis, VEGF, and VEGFR are increased in CRC, compared to normal mucosa and adenoma, and are further related to more malignant features of CRC including poorer survival.

### Inflammatory infiltration in CRCs

There are two types of immune responses, innate and adaptive immunity. Innate immunity reacts rapidly to molecular patterns found in microbes, independent of prior contact with a pathogen. The adaptive immune response is specific and has immunologic memory [81]. Immune responses play a critical role in host defence against many kinds of diseases including tumours. In immune responses against tumours, antigen-specific receptors presented on lymphocyte surface membranes recognize and specifically bind to the surface components of the tumour cell [82]. Tumour inflammatory infiltration (TII) mainly includes T cells and B cells (the adaptive response), as well as tumour-associated macrophages (TAM), dendritic cells (DCs), natural killer (NK) cells, neutrophils, mast cells and eosinophils (the innate response). The majority of TII cells are T cells, specifically CD4+ and CD8+, and the anti-tumour effects of T cells are considered to be mediated by cytokine secretion [83-85]. B-lymphocytes proliferating in the draining lymph node migrate into the tumour where they undergo further rounds of antigen-driven stimulation and proliferation, resulting in antibody secretion. The antibodies bind to tumours resulting in tumour destruction via phagocytes in the presence of complement. NK cells are another group of lymphocytes, and lack B-cell and T-cell receptors. NK cells are designed to kill certain mutant cells and virus-infected cells, by releasing proteolytic enzymes called granzymes, pore-forming proteins called perforins and chemokines. Granzymes pass through the pores and activate the enzymes that lead to apoptosis of the infected cells by means of destruction of their structural cytoskeletal proteins and by chromosomal degradation. As a result, the cells break into fragments that are subsequently removed by phagocytes. Perforins can also sometimes result in cell lysis. TAMs derive from circulating monocytic precursors, and are directed into the tumour by chemoattractant cytokines called chemokines. Tumour cells also produce cytokines that can prolong the survival of TAMs [86]. TAMs can kill and phagocytose tumour cells and remove apoptotic and necrotic tumour cells [87] by secreting lytic enzymes such as lysosomal enzymes, TNF-α and macrophage activation factor [82,88]. TAMs can also serve as antigen presenting cells, which evoke a strong immunologically mediated response [86,87,89]. DCs are a unique group of white blood cells and are present in a basically immature state. After taking up and processing antigen, DCs migrate to the lymphoid tissues where they interact with T cells and B cells to initiate and shape the immune response. DCs also activate non-specific effectors such as macrophages, NK cells and eosinophils [90].

The TII response may have dual effects in the development and progression of the tumour. On one hand, inflammatory cells can kill tumour cells, resulting in tumour regression and a greater chance of survival for the cancer patient. On the other hand, production of cytokines and growth factors derived from TII can stimulate tumour cells to grow and emigrate. The effects on tumour development may depend on host- and tumour-specific features such as the immunoresponse of the host or the type and biological features of the tumour [91-93]. For example, TAMs have tumour inhibitory effects as mentioned above, but also have tumour promoting effects. TAMs produce growth and angiogenic factors such as TNF-a, IL-1 b, IL-8, fibroblast growth factor, VEGF, epidermal growth factor [94] as well as protease enzymes which degrade the tumour ECM. Hence, TAMs stimulate tumour-cell proliferation, promote angiogenesis, and favour invasion and metastasis.

#### Lymphocytic infiltration in relation to clinicopathological variables

Many studies have shown that a high grade of lymphocytic infiltration or TII in CRC is related to favourable survival of patients [95-106], and the prognostic significance of the TII still remains even after adjustment for other clinicopathological variables in the whole group of CRC patients [107-115], patients with T1-2N0M0 or T3N0M0 CRC [89], with T1N0-3M0 or T1-4N1-3M1 [116], or stage II-III [44]. In addition, an extensive TII is related to better differentiation of tumours [99], earlier stage [99,110,116,117], lower rates of recurrence [96,103] and distant metastasis [103]. There are several possible explanations for the TII in relation to better survival and less malignant features in cancer patients. Firstly, the TII could represent a specific response by the host against the tumour. Secondly, the TII may function as a barrier for tumour penetration [112], and thirdly, tumours with extensive TII may respond better to chemotherapy. Our recent study showed that younger patients have more TII around rectal cancers, suggesting that younger patients had a better immunological response than older ones (unpublished data). However, there are controversial reports in which TII is not related to tumour differentiation, stage [97-117] or patient survival in CRCs [118]. In addition, TII is not associated with patient' gender, tumour location, or growth pattern [97-110]. One study shows that CD8+ T cell and macrophage infiltration are negatively related to the depth of invasion and vascular invasion [100].

Notably, TII in the inner part of the tumour is not significantly related to clinicopathological variables including patient survival, but abundant TII at the invasive margin of the tumour predicts a favourable prognosis in CRC patients [110]. These results indicate that TII at the invasive margin, compared with that in the inner part of tumour, is more effective against tumour development. Obviously, attention to the tumour invasive margin, not only to its morphology but also to its biology, is an important issue regarding tumour development and progression. Tumours with an infiltrative growth pattern at the invasive margin present a strong malignant phenotype and further predict a poor prognosis in CRC patients compared to tumours with a expansive growth pattern [66,119]. It has been observed that either expression of PINCH or phosphatase of regenerating liver (PRL) at the invasive margin of CRC is related to a poor prognosis, while their expression in the inner parts of the tumour is not [110,120]. The invasive margin is a critical area for stimulation of angiogenesis and lymphangiogenesis in tumours, which contributes to tumour invasion and metastasis. Dundas *et al*. have analysed 60 slides from 60 tissue blocks from 30 colonic carcinomas, and the slides are circulated twice to six histopathologists with varying experience [121]. Five out of the six pathologists showed a good to excellent intraobserver agreement for assessment of the character of the invasive margin, which is not significantly affected by sampling. The pathologists were not reliable in assessing peritumoural lymphocytic infiltrates, and this assessment was significantly affected by sampling. The results indicate that peritumoural lymphocytic infiltration is not a reproducible observation and may therefore not provide useful prognostic information in routine practice.

#### TAMs in relation to clinicopathological variables

TAMs are highly localized at the invasive margin of CRC compared with the central area of the tumour [44,122,123]. Furthermore, the number of TAMs in the invasive margin positively correlates with the degree of lymphocytes and apoptotic cancer cells [87,122,123]. CRC patients with a high TAM level have significantly less invasion in depth [100], lymph node [100,124] and blood vessel [100], and less local and distant recurrence [103,124]. Moreover, abundant TAMs are a sign for better survival of CRC patients [100,103]. In some studies, TAMs are even an independent prognostic factor of good survival [44,89]. One study shows that the presence of TAMs in regional lymph node metastases may serve as a predictor of better survival in patients with CRC of Dukes' stage C [125]. Taken together, the results suggest that TAMs are effective in inducing apoptosis of tumour cells and suppressing tumour spread at the front line of host defence, thereby inhibiting tumour development.

It has been shown that TAMs are positively associated with angiogenesis in CRC [94]. Such an association is also seen in liver metastasis from CRC, furthermore both TAMs and MVD independently predict worse prognosis [126]. However, some studies do not find associations of TAM numbers with microvessels [44,127], or any clinicopathological features including depth of invasion, stage, lymph node metastasis, vascular invasion, recurrence and prognosis [55,127]. FasL+ macrophages may also induce apoptosis of neighbouring Fas+ lymphocytes, which may explain a negative regulatory mechanism of TAMs against T cells distributed in the same areas [123,127].

#### DCs in relation to clinicopathological variables

Patients with CRC present reduced numbers of peripheral blood DCs compared with healthy controls [128]. Furthermore, the number of DCs is decreased in primary colon cancer compared with the normal colon mucosa [129] and is even less in lever/pelvic metastasis, six-fold lower than in primary CRC [130]. DCs are often present in the invasive margin of both primary and metastatic CRC [131-133]. DCs positively correlate with lymphocyte infiltration [131,132,134], but inversely correlate with levels of serum VEGF [128,135], probably because of the inhibition of VEGF on DC maturation in tumours [136]. Most studies in CRCs have shown that abundant DCs, frequently determined by DC markers of S-100, CD83 or CD86, are associated with less depth invasion [137], less lymph node involvement [137,138], less liver metastasis [128,137,138] or better survival [130,131,134,137,138]. A study performed on liver metastasis from CRC has shown that DCs determined by CD83 are positively related to apoptotic cancer cells, and independently predict a better prognosis [133]. These results indicate that DCs may act as one line of defence against tumour development of primary and metastatic CRC.

A study in 170 patients with rectal cancer using CD1a as a marker for DCs did not find that DCs are related to survival [48]. Notably, Sandel *et al*., using markers CD1a and CD208 for DCs in CRC, found that patients with high levels of either CD1a- or CD208-positive DCs had shorter survival [139]. However, the same group carried out another study on the same series of CRCs using the S-100 marker for DCs, and demonstrated that the presence of TAMs was a prognostic factor for better survival [134]. Since the same group studied the same patients using the same technique, the reason for the different results would not be due to sample error, method variation, or features of tumours or patients. It has been observed that CD83-positive DCs in the invasive margin form clusters with lymphocytes. Although the number of CD1a-positive DCs are almost the same as that of CD83-positive DCs in the invasive margin of the tumour, CD1a-positive DCs are mostly scattered and rarely form clusters with lymphocytes. DCs that express both CD1a and CD83 are rare [132]. The distinct infiltration pattern of DCs in tumours indicates various biological functions of DCs. In other words, these conflicting results regarding the role of DCs on CRC prognosis may partly depend on the different markers used in the different studies; for example, DCs that are determined by markers S-100 and CD83 often appear to be related to an immuno response with antitumour activity. Therefore, it could be of interest to study molecular issues of DC heterogeneity in order to identify unique biological functions in cancer development.

TII in CRC, especially at the invasive margin of tumours, plays a critical role against tumour development and aggressiveness, based on the relationship of strong TII with better differentiation, earlier stage, lower rates of local/distant recurrence and better survival.

### MMPs in CRCs

#### MMP expression and biological functions

Cell adhesion to the ECM is mediated by integrins. Focal adhesion (FA) is an integrin-rich cell adhesion sites, containing cytoskeletal signalling molecules including FA kinase, integrin-linked kinase (ILK), talin, vinculin and paxillin. ILK is an intracellular serine/threonine protein kinase regulating integrin-mediated cell adhesion, E-cadherin expression, pericellular fibronectin matrix assembly and cellular proliferation and survival [140-142]. Through FA a selective group of cytoskeletal and signalling proteins are recruited to cell matrix contact sites where they link the actin cytoskeleton to the ECM and where signals are transduced bidirectionally between the intracellular signalling network and the ECM [143]. The cell microenvironment and cell interactions with ECM play an essential role in many physiological and pathological processes. The ECM can actively regulate cellular proliferation, migration, adhesion and invasion, which influence embryonic development, tissue morphogenesis and angiogenesis as well as tumour transformation and metastasis. Tumour development is characterized by a severe aberration in the interaction of tumour cells with surrounding ECM.

During tumour progression, tumour cells must remodel the matrix either by expressing or degrading ECM proteins to facilitate communication and escape control by the microenvironment. The remodelling of the microenvironment surrounding tumour cells leads to the release of ECM-associated growth factors which may function to suppress or induce tumour growth [144]. Thus, many ECM-associated factors are proposed to be involved in the interaction of tumour cells with the ECM during tumour progression.

MMPs are a family of ECM degrading proteinases, secreted by both tumour and stromal cells. Based on substrate specificities and sequence characteristics, the classic MMP family members can be divided into at least four subgroups; collagenases, gelatinases, stromelysins, and matrilysins. So far, 23 different MMPs, MMP-1, -2, -3, -5, -7, -8, -9, -10, -11, -12, -13, -14, -15, -16, -17, -18, -19, -20, -21, -23, -25, -26, and -28, and four tissue inhibitors of metalloproteinases (TIMPs), TIMP-1, -2, -3 and -4, have been cloned [145-147]. MMPs play a major role in physiological and pathological processes such as embryonic development, differentiation, apoptosis, immune surveillance, wound healing, tumour angiogenesis and invasion and metastasis [148]. For instance, MMPs can cleave interleukin-2 receptor (IL-2R), an upregulator of T lymphocyte proliferation [149], and can activate TGF, an important inhibitor of the T-lymphocyte response against tumours [150], thereby suppressing the anti-tumour activity of T lymphocytes. MMPs are also important for endothelial invasion occurring during neovascularization. Application of a blocking peptide that prevents the interaction of MMP2 with its substrates has been shown to reduce angiogenesis. When tumour cells are introduced into MMP2 knockout mice, the tumours that develop are less vascularized and exhibit reduced growth compared to the tumours in wild-type animals [151].

Unlike classical oncogenes, MMPs are not upregulated by gene amplification or activating mutations, and the increased MMP expression in tumours is probably due to transcriptional changes. This might be the result of activation of oncogenes or loss of tumour suppressors. It has been demonstrated that MMP7 is upregulated by the transcription factor PEA3, and MMP1 and MMP13 are downregulated by the tumour suppressor p53 [152-154]. The enzymatic activity of the MMPs may be blocked specifically by TIMP-1 and TIMP-2. The balance between the MMPs and the TIMPs is thought to play a critical role in controlling ECM turnover and in maintaining matrix homeostasis.

#### MMPs in relation to clinicopathological variables

Among MMPs, MMP-1, -2, -3, -7, -9, -11, and -13 have been studied the most. Expression of MMPs, such as MMP-1, -2, -3, -7, and -9, is greater in CRC than in normal mucosa or in adenoma of the colon/rectum [155-167]. Higher levels of MMP-7 expression are also found in liver metastases compared with normal liver tissue [167]. However, there are a few reports showing inconsistent results on expression of MMPs in CRCs. For example, Roeb *et al*. have reported that expression of MMP-3 and -13 is greater in CRC but MMP-1 expression is not [165]. Bodey *et al*. have observed strong expression of MMP-3 and -10 in colon cancers but not MMP-13 [168]. Unlike other classical MMPs, MMP-19, -26, and -28 express in the normal intestine, but are downregulated in colon cancer. Thus, it has been proposed that they play a prominent role in tissue homeostasis [169].

Regarding the clinicopathological significance of MMPs, most findings have shown that increased expression of MMPs is related to more malignant features of CRCs. MMP-1 correlates with poor differentiation [155], advanced Dukes' stage, lymphatic invasion [155,161], hematogenous metastasis [155], and shorter survival [170]. MMP-2 expression is increased in CRC with infiltrative growth patterns compared with expansive growth patterns, and it has positive relationships with poor differentiation [171] and liver metastasis [172]. MMP-3 has been found to be associated with lymph node metastasis [159]. MMP-7 correlates with poor differentiation [163], depth of invasion, lymphatic involvement [95], advanced Dukes' stage [95,163], metastasis [95,173,174], and unfavourable survival [95]. Even in multivariate analysis, the prognostic significance of MMP-7 still remains [95,173]. MMP-9 is related to the presence of perineural invasion [159]. MMP-13 overexpression tends to predict a poor prognosis in patients with CRC [175]. Interestingly, Behrens *et al*. have found that hereditary nonpolyposis colorectal cancer syndrome (HNPCC) exhibited a significantly lower expression of MMP-1 and -9, whereas sporadic CRCs usually have increased expression of MMPs. These findings on the basis of lower matrix-degrading properties of the fibroblastic tumour stroma in HNPCC may help us to understand why HNPCC, compared with sporadic CRC, has lower malignancy, for example, a better prognosis in HNPCC patients [156].

During tumour invasion and metastasis, tumour cells must pass a series of basement membrane and ECM barriers. The basement membrane is the first and most important barrier for tumour cells to penetrate to complete invasive and metastatic processes. The ECM must be broken down to permit tumour cells to invade surrounding tissues or metastasise to other organs. Furthermore, some MMPs stimulate angiogenesis for promoting tumour growth and invasion. Although the mechanism by which MMPs enhance the invasive and metastatic competence of tumour cells seems straightforward, the specific role of distinct MMPs in the progression of tumour invasion and metastasis is more complex than has been assumed. For example, MMP-2 does not show an association with tumour differentiation, stage, metastasis, or patients' prognosis [157]. Oppositely, MMP-2 level in plasma is higher in T2 and T3 CRCs than T4 tumours [161]. MMP-9 expression has no link to either tumour stage or patients' survival [171]. CRC patients with overexpression of MMP-12 have a better prognosis compared with patients who do not show overexpression of MMP-12. Some of the MMPs, such as MMP-2, can convert plasminogen to angiostatin, which is a potent inhibitor of endothelial cell proliferation and angiogenesis [176,177]. Therefore, these MMPs may limit angiogenesis, inhibit tumour growth and suppress metastasis.

We found that ST3 (MMP11) is positively related to PINCH expression in CRC (unpublished data), and, both ST3 and PINCH are present in stromal fibroblasts around tumour cells but not in tumour cells themselves [178,179]. The ST3 gene is localized to chromosome 22q11.2, with 8 exons and 7 introns [180,181]. The term "stromlysin-3" is chosen because the protein has the same four-domain structure as previously described stromlysins and because "stromlysin" is correlated with ST3 RNA expression in stromal cells of breast cancer [182]. ST3 belongs to a new MMP subfamily according to its gene location, the sequence of the putative ST3 catalytic domain, and function. ST3 differs from those reported MMP genes on chromosomes 11, 16, and 19 [182], as the ST3 prodomain contains an additional recognition site for convertase-like enzymes such as furin. Consequently, the ST3 proenzyme, unlike other MMPs, is processed intracellularly and released as a mature enzyme [183]. Unlike most of the MMPs, which are activated outside the cell by other MMPs or serine proteinases, ST3 can also be activated inside the cell by intracellular furin-like serine proteinases [151,184].

Although the exact mechanism of ST3 action is unknown in tumour development, one hypothesis is that ST3 is implicated in basement membrane remodelling through release or activation of growth factors or cytokines stored in the ECM. ST3 degrades insulin-like growth factor-binding protein-1 (IGFBP-1), leading to cellular proliferation and survival [185]. It has been shown that cancer cells injected into *ST3*-null mice have an increased frequency of apoptosis and necrosis compared to the wild-type hosts [186]. ST3 inhibiton of apoptosis may be through the release of survival factors such as insulin-like growth factor (IGFs) [187]. ST3 is also involved in the escape mechanisms of TII, and decreases the sensitivity of tumour cells to NK cells [178], but positively regulates tumour angiogenesis [188]. Thus, ST3 may play a role in favouring cancer cell survival in the stromal environment during tumour development.

ST3 protein is present in the stromal fibroblasts around tumour cells but not in tumour cells themselves. ST3 expression, determined by Northern blot, in-situ hybridisation or immunohistochemistry, is undetectable in normal colorectal mucosa, detectable in low levels in adenoma, and at higher levels in primary CRC [178,189-194] and in metastasis of the lymph node and the liver. However, there is no significant difference in the levels of ST3 expression between the primary and metastatic tumours [178,189,193], or between the inner part and invasive margin of CRC. This indicates that a high degree of ECM turnover also takes place at the inner part of the tumour, and not only at the invasive margin where tumour cells promote invasion into surrounding tissue [190].

**Figure 1:**
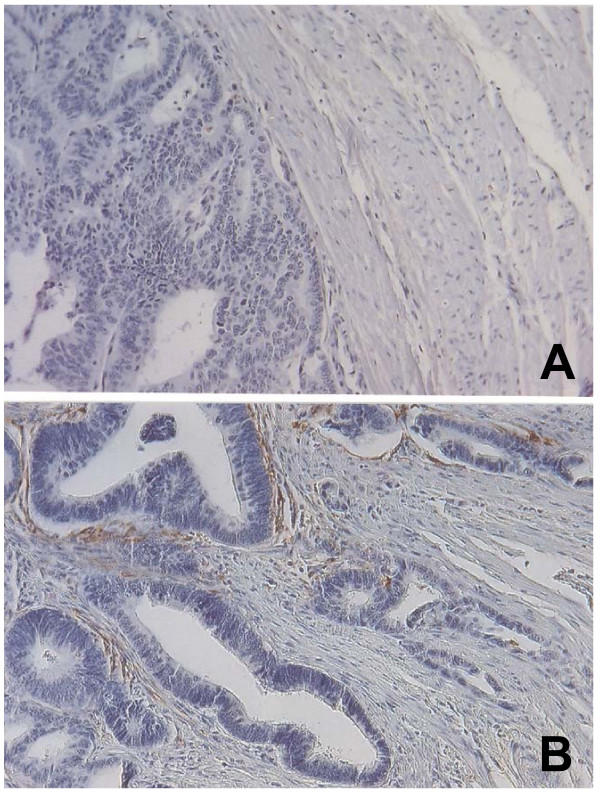
Expression of ST3 in primary colorectal adenocarcinomas by immunohistochemistry performed on paraffin-embedded tissue sections counterstained with hematoxylin: A) stromal cells of a tumour with an expanding growth pattern show negative expression, and B) stromal cells of a tumour with an infiltrative growth pattern show positive expression.

The rate of extensive ST3 expression is significantly higher in the de novo group than in the ex adenoma group. Histopathologically, the de novo group has a significantly higher rate of cases with an infiltrative invasion pattern. These results may indicate that ST3 expression is implicated in a greater invasive potential of CRC [192]. As shown in Figure 1, tumour with an infiltrative growth pattern has higher ST3 expression than that with an expanding growth pattern [178,194]. However, ST3 is highly expressed in Dukes A+B tumours compared to Dukes C+D tumours [178]. Taken together with similar levels of ST3 expression between the primary and metastatic tumours, it seems that ST3 is involved in the local invasion and early development of CRC, but is not a critical factor in the late stage of CRC development although one study shows that high expression of ST3 transcripts correlate with the progression of CRCs toward liver metastasis [189]. Another study shows that ST3 expression is higher in women than in men, and in distal tumours than in proximal tumours in CRC [178]. ST3 expression is not related to age, grade of differentiation, TII, the degree of tumour invasion, metastases or survival in CRC patients [178,190].

The MMP family is likely to be involved in early development of CRC via remodelling of the basement membrane, inhibition of tumour cell apoptosis and the host immune response, and angiogenesis activation. MMPs also play critical roles in tumour aggressiveness based on their relationships with advanced tumour stage, high frequency of recurrence and worse prognosis. However, MMPs can also suppress tumour growth, invasion and metastasis.

### PINCH expression in CRCs

#### PINCH expression and its biological functions

PINCH was originally identified by Rearden in 1994 from screening a human cDNA library with antibodies recognizing senescent erythrocytes [195]. The PINCH gene is located on chromosome 2q12.2, and encodes a 38 kDa protein. PINCH is an evolutionarily conserved adapter protein and has five LIM domains [195-199]. Adapter proteins, a group of non-catalytic proteins, are involved in specific protein-protein interactions, which mediate essential cellular processes including cellular proliferation, differentiation and survival by controlling signal transduction pathways. The LIM domain is a protein binding motif consisting of a cysteine-rich consensus sequence of approximately 50 amino acids folding into a specific three-dimensional structure comprising two zinc fingers. LIM domains are present in nuclear and cytoplasmic proteins that are essential for embryonic development and are involved in many pathological processes including tumourigenesis [198,200-202].

PINCH interacts directly with ILK through its LIM1 domain binding to the first of four ankyrin (ANK) repeat domains at the ILK N-terminus. The C-terminal domain of ILK has certain homologies with the catalytic domains of serine/threonine protein kinases. This kinase-like domain is able to interact with several components of cell-matrix contact sites including CH-ILKBP (α-parvin, actopaxin), β1, β2, and β3 integrin cytoplasm tails, β-parvin (affixin), and paxillin [203-207]. PINCH, ILK and CH-ILKBP form a ternary complex that can interact with other components of the cell-ECM adhesion structures via multiple mediated interactions and therefore play crucial roles at ECM adhesion sites [208]. The importance of this complex has been emphasized by a number of research groups. For example, overexpression of the N-terminus of PINCH or the N-terminus of ILK results in retarded cell spreading and reduced cell motility. The interaction of PINCH and ILK is crucial for cell shape regulation and migration via integrin activation. Inhibition of formation of the PINCH-ILK-CH-ILKBP complex leads to a significant reduction in fibronectin matrix deposition and inhibition of cell proliferation [209,210].

PINCH can also bind to Nck2, an additional PINCH binding partner, through the LIM4 domain of PINCH and the SH3 domain 3 of Nck2. Nck2, as an SH2/SH3 adaptor protein, is an important component of the signalling pathways of growth factor receptors including epidermal growth factor (EGF) and PDGF receptors, and can modulate actin dynamics by interacting with p21-activated kinase. Therefore, PINCH, by mediating the formation of the complex between ILK and Nck-2, is involved in the regulation of ILK function, which is implicated in many critical physiological and pathological processes [179,197,209-211].

Zhang *et al*. identified a second member of the PINCH family, PINCH2, and therefore, PINCH was renamed to PINCH1 [208]. The PINCH2 gene has been mapped to chromosome 2q14.3 and encodes a 39 kD protein. The PINCH2 protein also contains five LIM domains and has an overall similarity of 92% to PINCH1. At the embryonic stage, PINCH1 is expressed in the heart, lung, kidney, liver, thymus, spleen, bladder, stomach, intestine, skeletal muscle and facial regions especially surrounding skeletal structures, while PINCH2 expression is restricted to the bladder, stomach and intestine. In the intestine, PINCH1 expression is localized to epithelial cells and the smooth muscle layer, whereas PINCH2 expression is confined to the smooth muscle layer. In addition, high expression of PINCH1 is present in megakaryocytes during fetal liver hematopoiesis, where PINCH2 expression is undetectable. Megakaryocytes also express ILK and Nck2, the known binding partners of PINCH1. In adults, both PINCH1 and PINCH2 are expressed in the heart, lung, kidney, liver, bladder, uterus, testis, skin, skeletal muscle, large intestine and fat. In the spleen and thymus, only PINCH1 transcripts are present. Similarly to the embryonic intestine, PINCH1 expression is observed in the epithelial cell layer of the intestine and in the surrounding smooth muscle cells, whereas PINCH2 is confined to the smooth muscle layer [200].

PINCH2 also localizes to cell-ECM adhesion sites but only the LIM1 domain binds to ILK, suggesting that PINCH2 may potentially interact with other components of the cell-ECM adhesion structure. In addition to regulating the PINCH1-ILK interaction, cell spreading and migration, PINCH2 may participate in the regulation of nuclear processes since PINCH2 is present at high levels in the nucleus of the cell [200]. However, PINCH2 does not bind to Rsu-1 as PINCH1 does. Rsu-1 is a highly conserved leucine-rich repeat protein and expresses in various mammalian cells. Ectopic expression of Rsu-1 inhibits anchorage-independent growth of Ras-transformed cells and some human tumour cell lines [196].

#### PINCH in relation to clinicopathological variables

**Figure 2:**
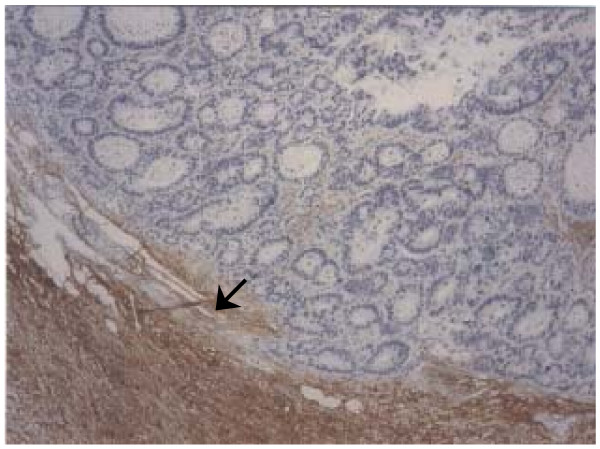
Expression of PINCH in primary colorectal adenocarcinoma by immunohistochemistry performed on a paraffin-embedded tissue section counterstained with hematoxylin: expression of PINCH protein at the invasive margin (arrow) was much stronger than in the inner tumour area.

After identifying the PINCH gene, Rearden's group in 2002 further analysed PINCH protein expression determined by immunohistochemistry using a polyclonal antibody against PINCH in human tissues, and observed that PINCH expression was markedly upregulated in the tumour-associated stroma of many common cancers including breast, prostate, lung, skin, and colon cancers, compared to the corresponding normal tissues [211]. As shown in Figure 2, PINCH is noted to be especially abundant in stromal cells at the invasive margin of the tumour, a region where signalling in the integrin and growth factor pathways is known to occur. Recently, we have further studied the clinicopathological significance of PINCH expression in a large series of CRCs by immunohistochemistry using the same antibody used in the above study. Our results show that the expression of PINCH protein in the stroma is not only increased in primary tumours compared to normal mucosa, but is also significantly increased in lymph node metastasis compared to primary tumours, and is more intense at the invasive margin than in the intratumoural stroma. Strong PINCH expression at the invasive margin of primary tumours is further related to lymph node metastasis and predicts a worse outcome in the patients, independent of Dukes' stage, growth pattern and grade of differentiation [179,212].

The intensity of PINCH expression in the inner part of primary tumour is not significantly related to survival and other clinicopathological variables including Dukes' stage, growth pattern and grade of differentiation [179]. Localization of the PINCH protein seems to be very critical for its function in tumour development and aggressiveness. That is, PINCH expression at the tumour invasive margin, but not in the inner part of the tumour, plays an important role in tumour aggressiveness.

The PINCH protein is detected not only in fibroblasts, but also in myofibroblasts and in a proportion of endothelial cells of the tumour vasculature (whereas normal epithelial and tumour cells do not show any staining) supporting the involvement of PINCH in promoting tumour-stromal interactions that support tumour progression [179,212]. PINCH expression has been found to be oppositely associated with TII, suggesting that the upregulation of PINCH may be the tumour-activated reaction against TII, leading to tumour progression. Myofibroblasts have been considered to be associated with desmoplastic stromal tumour responses, and proposed to form a barrier to the migration of immunocompetent cells toward the tumour and hence to reduce immune surveillance. TII is known to reflect of the tumour-associated immune response and is generally considered to be cytotoxic for the tumour cells [95,116]. A previous study in colon cancer showed a negative correlation of the presence of myofibroblasts with TII [213]. The presence of PINCH in endothelial cells of the tumour vasculature suggests that the PINCH protein is upregulated in tumour angiogenesis, which is particularly important and indispensable for tumour growth and metastasis.

The adapter protein PINCH, a new component of the cell-ECM adhesion structure, may have an important role in tumour invasion and metastasis, via tumour-stromal interactions, resulting in a poor prognosis. It is of interest to further study the biological and clinicopathological significance of PINCH 1 and PINCH2 in tumours.

### Stroma and cancer therapy

Although conventional chemotherapy and radiotherapy have improved the outcome for CRC patients, the benefits of the treatments are still under investigation, especially in patients with advanced-stage tumours. The side effects of the treatments and resistance to the treatments are still major problems. Because of a lack of specific markers to select patients for suitable treatments many patients have been overtreated by chemotherapy and radiotherapy. Thus, biomarkers for both tumour cells and stroma are urgently needed to complement current tumour stage in terms of response to treatments. Compared to tumour cells, the stroma has been shown to be a more attractive therapeutic target. Firstly, regulation of stromal activity could affect tumourigenesis in different ways including inhibition of angiogenesis, lymphangiogenesis and MMP activity as well as activation of certain TII, to stabilize and regress the primary tumour. Secondly, some stromal factors, such as endothelial cells, are highly accessible to circulating drugs or drug carriers [214]. Thirdly, the optimal dose for conventional cytotoxic anticancer agents has usually been defined as the maximum tolerated dose. In contrast, biological and antiangiogenic agents may achieve maximum therapeutic effect at doses below the maximum tolerated dose. Therefore, it is important to assess quantifiable effects on the molecular target or biological parameters downstream from the molecular target, as well as safety end points to establish the dose-effect relationship and determine both the optimal biological dose and the maximum tolerated dose [3]. Fourthly, stromal cells, compared to tumour cells, are less likely to develop drug resistance; although some stromal proteins are tumour-derived, most stromal proteins are the products of stromal cells. Finally, one of the major problems with conventional radio- or chemo-therapies is that they indiscriminately affect growing normal and tumourigenic tissue; therefore, a therapy targeted to the stroma would minimize the side effects of anti-cancer therapy [8].

#### Antiangiogenic therapy

Antiangiogenic therapy is a new promising strategy for inhibiting tumour growth, development and metastasis. A number of potential angiogenic inhibitors have been developed to affect endothelial cell proliferation, migration and survival, and some of these agents have entered clinical trials in CRC patients. PTK/ZK acts on VEGFR-1, -2 and -3, SU 11248 on VEGFR-1, -2, -3 and PDGFR, ZD 6474 on VEGFR-1, -2, -3 and EGFR, BAY 43–9006 on VEGFR-2, -3 and PDGFR, AEE 788 on VEGFR-1, -2 and EGFR, Imatinib on PDGFR, and gefitinib and erlotinib on EGFR [215]. Among the VEGFR family, the VEGFR-2/kinase-insert-domain containing receptor is upregulated during tumourigenesis. An anti- kinase-insert-domaincontaining receptor antibody, IMC-1C11, blocks VEGFR- kinase-insert-domain containing receptor interaction, and inhibits VEGFR-induced endothelial cell proliferation. There are two new monoclonal antibodies, bevacizumab (Avastin) targeting VEGF, and cetuximab targeting EGFR, which have been used for treating CRC patients with metastasis. Bevacizumab has been shown to improve progression-free survival and overall survival of metastatic CRC patients for both first-line and second-line combined treatments with irinotecan, fluorouracil/leucovorin or oxaliplatin [215]. It has been demonstrated that bevacizumab increases the activity of fluorouracil/leucovorin in the first-line treatment. Cetuximab directly inhibits EGFR by binding its extracellular region and blocking ligand-receptor interaction, thus preventing downstream signalling events. Data from several phase II trials have shown that the combination of cetuximab with fluorouracil/leucovorin plus irinotecan or fluorouracil/leucovorin plus oxaliplatin with irinotecan leads to a high response rate, a long time to progression, and a good prognosis in the first-line treatment of metastatic CRC. Panitumumab, a monoclonal antibody against EGFR, has also shown to be active in irinotecan and oxaliplatin-refractory metastatic CRC [216]. The accumulated data from preclinical experiments and clinical trials have led to the design of trials look at the activity of angiogenic inhibitors in combination with cytotoxic regimens, with the hope of further improving the outcome for patients with metastatic CRC [216].

#### Immunotherapies

Immunotherapies, as less toxic treatment modalities, have emerged as potentially attractive alternatives for cancer therapy. It has been shown that when patients with resectable recurrent CRC are treated with interleukin-2 before surgery, there is an increase of eosinophilic infiltration in tumour tissue, indicating that interleukin-2 increases the host response to the tumour [217]. In another study, OK-432, an immunomodulatory agent prepared from an attenuated strain of Streptococcus pyogenes, was injected intratumourally in patients with CRC. Postoperative examination revealed the formation of fibrin fibers at the site of injection, and further marked TII cell infiltration including many giant cells in the tumour stroma, leading to extensive regression of the tumour [218].

### MMPIs

MMPIs, such as BAY12–9566, AG3340, BMS275291 and CGS27023A/MPI270, have been shown to inhibit tumour growth in preclinical models. However, this treatment has achieved minimal success in patients with advanced cancers in clinical trials. The differences between preclinical and clinical results with MMPIs are not just due to differences between animal and man but are rather related to the stage of disease, different endpoints, and the treatment methods. For example, MMPIs are unlikely to be effective in patients with advanced-stage cancer. However, there are significant preclinical data to support a role for MMPIs in earlier stages of cancer. In addition, MMPIs are used to treat patients with endpoints of increased time to progression or improved outcome. In comparison, preclinical experiments performed in animals is to examine tumour progression with the final endpoints of reduced tumour number and size or metastasis. Finally, patients are given the maximum tolerated dose and this is often limited by musculoskeletal side-effects while animal studies used escalating doses of MMPIs that are not limited since mice are less susceptible to this side-effect. Thus, it is of importance to design MMPIs for selected patients. It is also important to select MMPIs-1, -3, -7, -9, and -13 as potential agents, since the expression of the corresponding MMPs correlates with metastasis and poor prognosis in cancer patients. Moreover, MMPs have a dual function in tumour development; namely, they play critical roles not only in tumour aggressiveness but also in suppression of tumour growth. This complicates treatment targeting MMPs. Based on accumulated data, it is recommended that future MMPI trials focus on: (1) patients with early stage cancer; (2) the use of MMPIs along with chemotherapy; (3) the measurement of MMPs in tumour tissue and blood as a means of identifying patients who are more likely to respond to MMPI therapy; and (4) identification of biomarkers that reflect activation or inhibition of MMPs in vivo [219].

MMPs have been suggested as biomarkers for selecting cancer patients for suitable treatments. Ogata *et al*. examined patients with CRC at stage II or III, who underwent potentially curative resection. The patients were divided into two groups: one group received postoperative administration of fluoropyrimidines (such as UFT and 5'-DFUR), and the other group underwent surgery alone. The disease-free survival rate in the chemotherapy group was significantly higher than that of the surgery-alone group. However, this difference was not seen between the two groups who had MMP-9 positive tumour. Thus, the efficacy of the chemotherapy may not be great for patients with a tumour positive for MMP-9 [220].

Although there are promising results in the treatment of cancer patients with immunotherapy, anti-angiogenesis and MMPIs, the roles and effects of these treatments are still under investigation. An important challenge is how to combine biological agents with different cytotoxic agents in CRC. Another important challenge is to combine therapeutic targets of tumour cells with those of stromal factors.

## Conclusion

**Figure 3:**
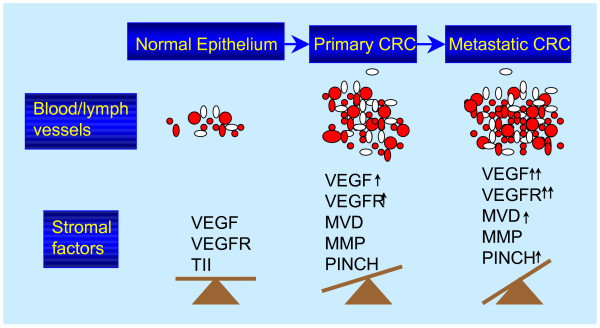
Alterations of tumour stromal factors during the development of CRC from the normal epithelium to primary CRC, and further to metastatic CRC. The number and construction of blood/lymph vessels, as well as the stromal factors, such as VEGF, VEGFR, MVD, MMP and PINCH are changed, which resulte in an imbalance of cell growth.

It has been suggested that CRC is caused by both environment, including life style, and genetic predisposition. In general, the number of genetic alterations is increased from the first genetic change in a normal endothelial cell to several genetic alterations in the late stages of cancer cells. Moreover, in the tumour stroma, the number and construction of blood and lymph vessels are altered by stimulation with many stromal factors such as VEGF, VEGFR, TII, MMP, PINCH, and others. Interactions between these growth and anti-growth stromal factors in the tumour stroma affect the formation, development and progression of CRC (Figure 3). Anticancer therapy targeted to the stroma is a promising strategy for inhibiting tumour progression.

## Abbreviations

CRC: colorectal cancer. DC: dendritic cell. ECM: extracellular matrix. FA: focal adhesion. HNPCC: hereditary nonpolyposis colorectal cancer syndrome. IGF: insulin-like growth factor. ILK: integrin-linked kinase. MMP: matrix metalloproteinase. MMPI: Matrix metalloproteinase inhibitor. MVD: microvessel density. NK: natural killer. PDGF: platelet-derived growth factor. PINCH: particularly interesting new cysteine-histidine rich protein. SMA: smooth muscle actin. ST3: stromelysin-3. TAM: tumour-associated macrophage. TII: tumour inflammatory infiltration. TIMP: tissue inhibitors of metalloproteinases. VEGF: vascular endothelial growth factor. VEGFR: vascular endothelial growth factor receptor.
